# Role of exosomes in gastrointestinal physiology and pathophysiology

**DOI:** 10.3389/fimmu.2025.1717977

**Published:** 2026-01-16

**Authors:** Vivian Naa Amua Wellington, Soudamani Singh

**Affiliations:** Department of Biomedical Sciences, Joan C. Edwards School of Medicine, Marshall University, Huntington, WV, United States

**Keywords:** epithelial barrier function, exosomes, immune modulation, gut, inflammatory bowel disease, intestinal pathophysiology, intestinal physiology

## Abstract

Exosomes, which are molecular cargo-containing, nanosized extracellular vesicles formed through double invagination of the plasma membrane, have emerged as important mediators of intercellular communication within the gastrointestinal tract. In addition to its established function in digestion and nutrient uptake, the gastrointestinal tract is central to immune regulation and maintenance of epithelial barrier integrity. Exosomes derived from intestinal epithelial cells, the gut microbiota and gut resident immune cells are key in sustaining intestinal homeostasis and regulating host-microbiota interactions. Dysregulation of these vesicles is increasingly linked to gastrointestinal disease pathogenesis, including inflammatory bowel disease. Currently, exosomes are being explored for use as diagnostic biomarkers and therapeutic agents in gastrointestinal ailments. In this review, we examine the roles of exosomes in gastrointestinal health and disease, highlighting their contributions in the regulation of epithelial barrier function, modulation of immune responses and communication with the gut microbiota. We further discuss the dysregulation of exosome-mediated signaling pathways in IBD and assess their potential as next-generation therapies for gastrointestinal disorders.

## 1 Introduction

The gastrointestinal (GI) tract is a continuous hollow tube extending from the mouth to the anus and forms a central component of the digestive system. It is essential for physiological processes such as ingestion, digestion, nutrient absorption and immune function (1). Within the gut ecosystem, heterogeneous cell populations coordinate the interaction of epithelial, immunologic, neural, and microbial cell networks in order to perform these crucial physiological functions (2). Thus, maintaining homeostasis in this complex environment relies heavily on intercellular communication. Notably, the GI tract is considered the body’s largest endocrine organ, reflecting its extensive role in systemic signaling pathways (3). While hormone-mediated intercellular communication in the gut has been substantially explored, extracellular vesicles (EVs) have emerged as key mediators of intercellular communication within the gut. EVs are cell-derived, membrane-bound vesicles that transport bioactive molecules between cells. These vesicles differ in origin and size and include apoptotic bodies, microvesicles, as well as exosomes (4). It is important to note that the ‘Minimum Information for Studies of Extracellular Vesicles’ (MISEV2023) guidelines recommend the use of the operational terms large EVs and small EVs “unless the subcellular origin can be demonstrated” (5, 6). However, use of previous terminology remains prevalent in current literature, including in studies that have not demonstrated subcellular origin. For consistency with existing studies discussed herein, the term exosome is used throughout this review.

**Figure 1:**
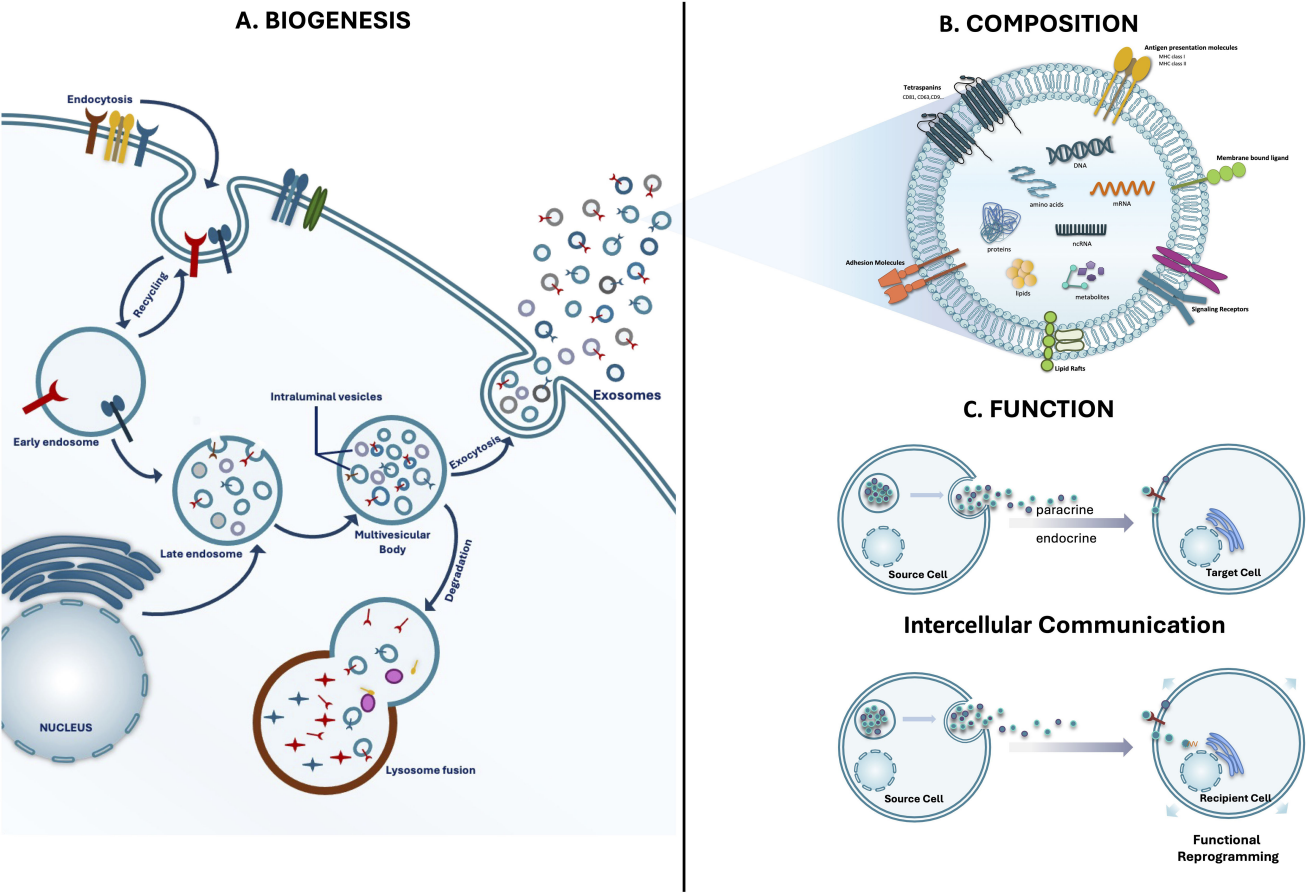
Exosomes as intercellular shuttles. This figure provides a comprehensive overview of exosomes as key mediators of intercellular communication. The left panel, **(A)** Biogenesis, illustrates the formation of exosomes, beginning with the invagination of the plasma membrane (endocytosis) to form early and late endosomes. These mature into multivesicular bodies (MVBs), which contain intraluminal vesicles. Finally, the MVBs fuse can fuse with lysosomes for cargo degradation or traffic to the plasma membrane, where they release the ILVs into the extracellular space as exosomes. The right panel, **(B)** Composition & **(C)** Function, details the diverse molecular cargo carried by exosomes, including proteins, lipids, enzymes, DNA, and various RNAs (mRNA and miRNA). These components facilitate different forms of communication: autocrine/paracrine signaling and the direct transfer of functional molecules to recipient cells, which can result in functional reprogramming of recipient cells.

Exosomes, the smallest of extracellular vesicles (30–150 nm in diameter), are secreted by diverse cell types into the extracellular environment (**Figure 1**). The sheer diversity of cell types that secrete exosomes as well as their presence in various biological fluids such as breast milk, urine, semen, saliva, plasma and gastric acid, strongly supports their physiological relevance (7–9). Once exclusively regarded as capsules of cellular debris, these nanovesicles facilitate the transfer of proteins, lipids, nucleic acids, and other bioactive molecules between cells. Through this cargo, they enable coordinated information transfer in an autocrine, paracrine, and endocrine manner (**Figure 1C**) (7, 10). Exosomal content can reflect the physiological or pathological state of their cells of origin and can significantly alter the function of recipient cells. Upon uptake, recipient cells may reprogram exosomal content to modulate vital physiological processes such as signal transduction, cell proliferation, apoptosis and immune activation (11, 12). Growing evidence has highlighted the pivotal role of exosomes in gut physiology and their involvement in gastrointestinal diseases (13–15). Consequently, exosome regulation of gut-relevant cellular processes continues to attract significant interest (16). Indeed, exosome dysregulation is associated with several GI disorders, including inflammatory bowel disease (IBD), colorectal cancer, and infectious enteric diseases. For example, aberrant exosome production by immune and epithelial cells contributes to tissue damage and persistent inflammation in IBD (17). In colorectal cancer, tumor-derived exosomes facilitate immune evasion, tumor development, and metastasis (18). At the same time, exosomes hold significant clinical potential as diagnostic biomarkers and targeted therapeutic agents for the management of gastrointestinal disorders. Their inherent biocompatibility, ability to traverse biological barriers, low toxicity and limited immunogenicity make exosomes particularly well-suited for therapeutic drug delivery applications (19).

Here, we summarize the critical function of gut-derived exosomes in maintaining gut epithelial barrier integrity, regulating immune responses, and facilitating host-microbiota communication. We also examine how disruption of these mechanisms influences the pathogenesis of IBD and evaluate the potential of exosomes as diagnostic biomarkers and therapeutic agents. By shedding light on these processes, this review aims to enhance understanding of exosome-mediated mechanisms in gastrointestinal pathobiology and inform future strategies for diagnosis and treatment.

## 2 Biogenesis of exosomes:

Exosome biogenesis (**Figure 1A**) is a tightly regulated process that originates within the endosomal system (20). It begins with the formation of early endosomes through inward budding of the plasma membrane (endocytosis). Early endosomes may be recycled back to the plasma membrane or mature into late endosomes. During maturation, the limiting membrane of endosomes further invaginates, encapsulating cytoplasmic components to generate intraluminal vesicles (ILVs) (21). Late endosomal structures containing multiple ILVs are known as multivesicular bodies (MVBs) (22). MVBs then follow one of several fates: they may be directed to the trans-Golgi network for recycling, fuse with lysosomes for degradation, or be trafficked to the plasma membrane, where they release their ILVs as exosomes into the extracellular space (23). Two primary mechanisms govern ILV formation: the endosomal sorting complex required for transport (ESCRT)-dependent and ESCRT-independent pathways (21, 24).

The ESCRT machinery consists of four distinct multimeric protein complexes (ESCRT-0, ESCRT-I, ESCRT-II, and ESCRT-III) along with associated proteins such as the AAA ATPase VPS4. These complexes are sequentially recruited to endosomal membranes to coordinate the selective sorting and packaging of cargo into ILVs (25, 26). ESCRT-0 recognizes and sequesters ubiquitinated cargo within phosphatidylinositol-3-phosphate-enriched endosomal compartments. ESCRT-I and ESCRT-II are then recruited to induce membrane curvature and initiate bud formation. ESCRT-III, together with VPS4, mediates the scission of ILVs from the endosomal membrane and subsequent dissociation and recycling of the ESCRT-III complex (27). Scission completion results in the formation of MVBs containing ILVs loaded with specific cargo molecules. Alternative ESCRT-dependent mechanisms involving accessory proteins such as Alix, HD-PTP, and Tsg101 have also been described (24, 28).

Evidence for ESCRT-independent exosome biogenesis pathways have been reported and reviewed in detail (29–31). Briefly, these pathways rely on distinct lipid- and protein-mediated mechanisms. One notable mechanism involves ceramide-induced membrane budding and cargo sorting. Here, ceramide, generated by the enzymatic activity of neutral sphingomyelinase, promotes negative membrane curvature, facilitating ILV formation.

Lipid rafts, which are cholesterol- and sphingolipid-rich microdomains within endosomal membranes, also contribute to organizing associated proteins such as caveolin-1 and flotillins, which assist in cargo sorting and ILV budding (29). In parallel, tetraspanins, a family of integral membrane proteins enriched in exosomes, represent another ESCRT-independent mechanism. Tetraspanins such as CD9, CD63, CD81, and CD82 (29, 32, 33) may organize into tetraspanin-enriched microdomains that contribute to cargo sorting, vesicle formation, and membrane fusion events that facilitate exosome release. Notably, ESCRT-dependent and -independent pathways may overlap (34), resulting in the formation of heterogeneous populations of ILVs within a single MVB and highlighting the adaptable nature of exosome biogenesis.

## 3 Exosomes in gastrointestinal physiology

**Figure 2:**
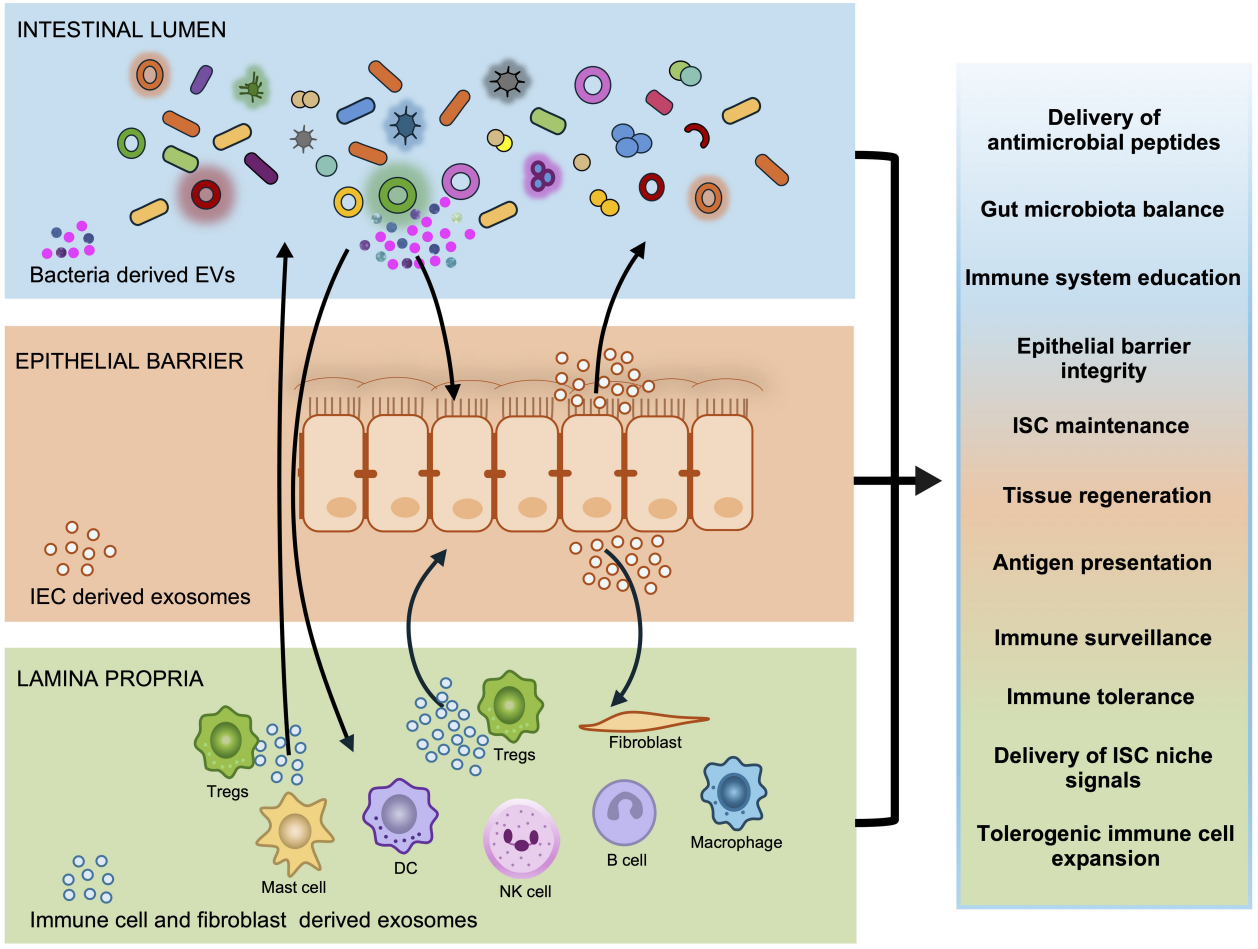
Exosomes in maintaining gut homeostasis. Exosomes are central to maintaining gut homeostasis by mediating crucial communication across the intestinal lumen, epithelial barrier, and lamina propria. Bacteria exosomelike vesicles (BEVs) released by the gut microbiota in the intestinal lumen may cross the epithelial barrier and can influence the host. Concurrently, Intestinal Epithelial Cells (IECs) secrete exosomes both apically into the lumen and basolaterally into the lamina propria. These exosomes play a vital role in immune surveillance, healing and strengthening the epithelial barrier. Exosomes from immune cells and fibroblasts within the lamina propria also engage in this complex crosstalk. These interactions lead to the modulation of immune responses, promoting immune tolerance and anti-inflammation. Together, these exosome-mediated interactions ensure gut homeostasis.

In the normal gut, mucosal homeostasis depends on the ability of the local immune system to maintain tolerance toward commensal microbiota while mounting efficient immune responses to eliminate enteric pathogens (35–37). Exosomes facilitate crosstalk between intestinal epithelial cells (IECs), immune cells, and the gut microbiota (38) (**Figure 2**). Hence, they are key players in sustaining normal physiological process within the gut.

### 3.1 Exosome-mediated regulation of epithelial homeostasis and barrier function:

The intestinal epithelium is a selective physical and biochemical barrier that separates luminal contents from the underlying tissue, protecting against microbial invasion and preserving tissue homeostasis (39). It is composed of a single layer of polarized columnar epithelial cells interconnected by tight junctions (TJs) and covered by a protective mucosal layer (40). Together, these features maintain a crucial separation between the host tissue and the gut microbiota, preventing microbial translocation and supporting immune tolerance (41). Disruption of TJs is often driven by an imbalance in cytokine signaling, particularly an excess of pro-inflammatory cytokines, which compromises barrier function and increases epithelial permeability, a common feature of many gastrointestinal disorders. Conversely, anti-inflammatory cytokines like IL-10, and transforming growth factor (TGF-β) play a protective role, preserving TJ integrity and promoting mucosal healing (42). Gut-derived exosomes may interact with various immune cell populations within the intestinal microenvironment. Through this interaction, and their bioactive cargo, they modulate immune responses and contribute to the maintenance of the epithelial barrier (21, 43).

Intestinal epithelial cells secrete exosomes. Van Niel et al. (38) demonstrated that IECs actively release exosome-like vesicles. These epithelial-derived exosomes were found to contain several key molecules, including MHC class I, MHC class II, CD68, CD63, CD26, and the A33 antigen (38, 44, 45). These findings suggested that IEC-derived exosomes participate in antigen presentation, potentially impacting mucosal and systemic immunity without necessitating direct contact between IECs and immune effector cells. Indeed, IECs can capture luminal antigens, package them into exosomes, and export this complex to local professional antigen-presenting cells (APCs) thus amplifying antigen presentation and shaping adaptive immune responses (46). Specifically, dendritic cells (DCs) exposed to IEC-derived exosomes laden with αvβ6/ovalbumin antigen produced active TGF-β, whereas DCs treated with antigen alone secreted only latent TGF-β. *In vivo* administration of these exosomes also promoted the development of antigen-specific regulatory T cells, while suppressing Th2-driven responses to food antigens (47). These findings highlight a critical immunoregulatory role for IEC-derived exosomes in enhancing peripheral tolerance and shaping mucosal immune responses. Moreover, IEC-derived exosomes originate from multiple epithelial subtypes, including enterocytes, goblet cells, Paneth cells, enteroendocrine cells, tuft cells, progenitor cells, transient-amplifying cells, and stem cells (48, 49). This diversity is reflected in the complexity of the exosomal cargo and its roles in regulating intestinal barrier integrity and mucosal homeostasis.

IECs secrete exosomes in a polarized fashion both apically into the intestinal lumen and basolaterally into the lamina propria (50). Apical exosome secretion supports host defense by delivering antimicrobial peptides such as cathelicidin LL-37 and β-defensin 2 (51), which bind and neutralize pathogenic bacteria, thus contributing to innate immune defense and reinforcing epithelial defense (51). During *Cryptosporidium parvum* infection, Toll-like receptor (TLR4) signaling-mediated suppression of the let-7 miRNA family increases the luminal release of these antimicrobial-laden exosomes, indicating that epithelial cells can adjust their exosomal output to augment innate immune responses to pathogens (51). Basolaterally secreted exosomes, on the other hand, are involved in immune surveillance and regulation. These vesicles can present antigens via MHC II molecules and carry costimulatory markers, functioning similarly to professional APCs. (38, 45, 46). One of the early demonstrations of this is from Karlsson et al.’s paper (52), which described “tolerosomes”, exosome-like vesicles secreted basolaterally by IECs, capable of inducing tolerance in naïve animals. Removal of these tolerosomes from circulation abrogated tolerance induction, emphasizing their immunological relevance.

IEC-derived exosomes are also enriched with anti-inflammatory and pro-resolving molecules. For example, transforming growth factor-beta 1 (TGF-β1)-containing exosomes that exhibit immunosuppressive properties were secreted by IECs under physiological conditions. These vesicles promoted regulatory T cells (Tregs) differentiation and the generation of tolerogenic DCs via ERK signaling, effects that were lost when EpCAM expression or exosome secretory pathways were disrupted (53). Complementarily, IECs generated tolerogenic exosomes enriched in IL-10 and antigen/MHC II complexes when exposed to a combination of ovalbumin antigen and the immunoregulatory peptide vasoactive intestinal peptide. These exosomes were able to convert antigen-specific CD4^+^ T cells into Tr1 regulatory cells, underscoring their significance in establishing antigen-specific immune tolerance (54). Leoni et al. (55) further showed that IECs secrete the endogenous pro-resolving mediator annexin A1 in exosomes, which promote mucosal wound healing by binding formyl peptide receptors and triggering epithelial repair.

IEC-derived exosomes play a pivotal role in regulating epithelial barrier function through the delivery of bioactive molecules involved in TJ dynamics. Exosomal RNAs can rapidly and locally remodel the extracellular matrix, regulate junctional proteins, coordinate immune recruitment, to maintain barrier integrity during injury and inflammation (40, 56). Exosomes from epithelial cell-derived miR-146a enhanced IL-10 production by monocytes (57), promoting an anti-inflammatory environment and protecting against ischemia/reperfusion injury by downregulating the pro-inflammatory TLR4/TRAF6/NF-κB pathway (58). Further, injured epithelial cells were found to release increased numbers of exosomes that stimulated fibroblast proliferation and upregulated α-smooth muscle actin, F-actin, and type I collagen. This activation was largely driven by the transfer of exosomal TGF-β1 mRNA as a rapid signaling mechanism for initiating tissue repair (59).

The intestinal epithelium undergoes continuous renewal through the proliferation and differentiation of intestinal stem cells (ISCs), located at the base of crypts. These stem cells rely on a sophisticated microenvironment of niche signals, including Wnt and epidermal growth factor (EGF) (60, 61). Wnt signaling regulates ISC maintenance, proliferation, differentiation, apoptosis, and migration (61). Wnts are secreted on the surface of exosomes which in turn activate Wnt signaling in recipient cells (62). Chen et al. (63) demonstrated polarity-dependent release of compositionally distinct Wnt-containing exosomes from epithelial cells, suggesting refined regional regulation of Wnt activity. Moreover, the exosomal marker A33 antigen, expressed predominantly in IECs, plays a role in epithelial migration and proliferation. A33-deficient mice exhibit defective wound healing and reduced epithelial cell turnover, underscoring the contribution of IEC-derived exosomes to mucosal repair (64). In addition to IECs, exosomes derived from fibroblasts, and other cell types within the gut mucosa and lamina propria also contribute to epithelial renewal. Oszvald et al. (60), using mouse and human intestinal organoids showed that intestinal fibroblast-derived small extracellular vesicles (sEVs) deliver EGF signals that support ISC niche function and organoid survival. Beyond local signaling, EVs may mediate long-range communication during tissue regeneration. Gurriarán-Rodríguez et al. (65) discovered the presence of an “exosome-binding peptide” motif within Wnts, required for their loading onto EV surfaces and showed that, following muscle injury, Wnt7a is secreted on EVs and transported over long distances, highlighting the systemic signaling potential of EV-associated Wnts. Similarly, Wnt5a, known to accumulate at epithelial wound sites has been detected in Caco-2 cell-derived exosomes, reinforcing the role of exosomes in epithelial repair after injury (66, 67).

Collectively, these findings demonstrate that IEC- and intestinal fibroblast-derived exosomes are multifaceted regulators of gastrointestinal homeostasis (Summarized in **Table 1**). They mediate immune modulation via antigen presentation and RNA transfer, support stem cell renewal through Wnt and EGF delivery, enhance antimicrobial defenses through apical secretion of antimicrobial peptides, and promote epithelial regeneration.

**Table 1:** Functions of intestinal epithelial cell (IEC) and fibroblast-derived exosomes in gastrointestinal physiology.

Function/source	Key cargo	Destination/target	Effect on gut physiology	Selected refs
Antimicrobial Defense/IECs	Antimicrobial peptides (LL-37, β-defensin 2)	Lumen/Microbes	Binds and neutralizes luminal pathogens. Reinforces innate immune defense.	(51)
Antigen Presentation & Immune Tolerance/IECs	MHC I/II, peptide complexes	Lamina Propria/DCs & Immune Cells	Antigen presentation to dendritic cells and other immune cells. Initiates and promotes immune surveillance.	(38, 45, 46)
	Tolerosomes; MHC II/Peptide complexes	Systemic/Immune Cells	Induction of “tolerosomes” that promote antigen-specific tolerance in naïve animals.	(52)
Anti-Inflammatory Cargo/IECs	TGF-β1	Lamina Propria/DCs & T Cells	Promotes Treg differentiation & generation of tolerogenic DCs. Creates anti-inflammatory environment.	(53)
	IL-10, Antigen/MHC II complexes	Lamina Propria/T Cells	Converts naïve CD4^+^ T cells into IL-10-producing Tr1 cells *in vitro*. Establishes antigen-specific immune tolerance.	(54)
Pro-resolving Cargo/IECS	Annexin A1	Epithelium	Binds formyl peptide receptors to trigger epithelial repair pathways. Promotes resolution of inflammation	(55)
	miR-146a	Monocytes, Epithelium	Downregulation of TLR4/NF-κB pathway; enhanced IL-10 production. Protects against ischemia/reperfusion injury	(57, 58)
	TGF-β1 mRNA	Fibroblasts	Activates fibroblasts, Initiates rapid tissue repair and regenerative responses.	(59)
Stem Cell & Epithelial Renewal/IECs, Fibroblasts	Wnt proteins	Intestinal Stem Cells	Activates Wnt signaling in recipient. Regulates ISC maintenance, proliferation, and differentiation.	(62, 63)
	EGF signals (from fibroblast sEVs)	Intestinal Stem Cells	Supports ISC niche function and organoid survival. Promotes epithelial renewal and regeneration.	(60)
	A33 antigen	Epithelium/Lamina propria	Role in epithelial migration and proliferation. Contributes to mucosal repair; wound healing.	(64)

DC, dendritic cell; IEC, intestinal epithelial cell; MHC, major histocompatibility complex; sEV, small extracellular vesicle; Treg, regulatory T cell.

### 3.2 Exosomes in immune regulation and gut homeostasis

Immune cells mediate response against pathogens while maintaining tolerance to harmless antigens. Dendritic cells and macrophages, as classical APCs, internalize antigens and present them via MHC class I and II molecules to CD8^+^ and CD4^+^ T cells, respectively, thereby initiating adaptive immune responses (68). Exosomes released by immune cells are important modulators of innate and adaptive immune responses. By transporting antigen-MHC complexes, delivering immunomodulatory cytokines, lipids and regulatory nucleic acids, these vesicles enable immune cells to coordinate immune activation and tolerance locally in the intestinal mucosa and systemically (69). In this section, we examine the significance of immune cell-derived exosomes in the initiation and modulation of immune responses in the context of maintaining gut homeostasis and immune tolerance.

#### 3.2.1 Mast cell-derived exosomes

Mast cells (MCs) release immunologically active exosomes (MC-EXOs) (70). These exosomes promote the activation of B and T lymphocytes and support the functional maturation of dendritic cells (71). By inducing potent antigen-presentation in dendritic cells, MC-EXOs facilitate robust T cell responses. They can drive Th1 polarization through stimulation of IL-12p70 secretion (72) and support Th2 differentiation via OX40L-mediated signaling (73), highlighting their ability to modulate immune polarization.

In addition to their immunomodulatory functions, MC-EXOs impact epithelial integrity through the transfer of regulatory RNAs. Wang et al. (74) demonstrated that exosomes from mast cells carried the long non-coding RNA (lncRNA) NEAT1, which acted as a molecular sponge for miR-211-5p. This released the repression of glial cell line-derived neurotrophic factor (GDNF), a protective factor for epithelial barrier integrity. MC-EXOs also interact with mast cells allowing for functional transfer of exosomal mRNAs and miRNAs and thus are conduits of indirect communication between mast cells (12).

#### 3.2.2 Dendritic cell-derived exosomes

Dendritic cells are potent APCs important in immune surveillance. Depending on their maturation state, they may steer immune response towards tolerance or activation. Generally, immature DCs promote T-cell tolerance, whereas mature DCs induce robust T-cell-mediated immune activation (75). This functional duality is reflected in the properties of dendritic cell-derived exosomes (DEX). While DEX are commonly rich in key immunological molecules such as MHC I and II alongside associated co-stimulatory proteins (76–78), exosomes derived from mature DCs express higher levels of these molecules, making them significantly more effective in activating T cells (79, 80). Segura et al. (81) demonstrated that exosomes from mature LPS-treated DCs were significantly more efficient in activating antigen-specific T cells *in vitro* and could endow B cells with the capacity to prime naïve T cells. *In vivo*, only mature DEX were able to induce effector T cell responses, with functional studies confirming that MHC II and ICAM-1 were essential for this priming effect.

Besides T cell interactions, DCs also use exosomes to communicate with neighboring DCs. Montecalvo et al. (82) showed that DEX carry distinct miRNA profiles depending on the maturation state of the parent DC. These exosomes can fuse with recipient DCs, delivering their miRNA content directly into the cytosol, thus unveiling a mechanism of DC-DC communication and post-transcriptional regulation via exosome-shuttled miRNAs. Furthermore, mature DEX may enhance the antigen-presenting capability of their parent DCs by stimulating increased expression of MHC I/II and co-stimulatory molecules such as CD40, CD54, and CD80 (80). In line with this, Sobo-Vuljanovic et al. (83) reported that DEX can assist in DC sentinel function by binding and cross-presenting bacterial TLR ligands to neighboring “bystander” DCs, promoting DC maturation, increased pro-inflammatory cytokine secretion, and natural killer (NK) cell activation.

In addition to their roles in immune activation, DEX can also mediate immune tolerance. Exosomes from immature DCs have been shown to suppress alloreactive T cell responses and promote Treg expansion, partly through the upregulation of IL-10 production in CD4^+^CD25^+^ Tregs, in models of intestinal transplantation (84). These tolerogenic vesicles are themselves enriched in immunosuppressive cytokines such as IL-10 and TGF-β (85), supporting Treg induction and dampening excessive immune activation (86). Similarly, exosomes derived from genetically modified DCs expressing FasL or those treated with IL-10 exhibit anti-inflammatory and immunosuppressive properties (87, 88).

#### 3.2.3 Macrophage-derived exosomes

Exosomes secreted by macrophages (MEX) display diverse immunomodulatory properties. The activation state of the macrophage is reflected in, and shapes, the molecular profile of their exosomes. LPS-stimulated RAW 264.7 macrophages release exosomes enriched in distinct cytokines and miRNAs compared to unstimulated cells. RNA sequencing of the exosomal contents from stimulated macrophages showed upregulation of genes linked to innate and adaptive immunity, including NF-κB signaling, TLR pathways, and MHC-mediated antigen presentation, collectively priming recipient cells for immune challenges (89). Under inflammatory conditions, MEX may deliver pro-inflammatory mediators such as TNF-α, IL-1β, and IL-6, contributing to immune activation and tissue damage (90). Conversely, MEX may transport anti-inflammatory and pro-fibrotic factors, including TGF-β, fibroblast growth factors (FGFs), granulocyte colony-stimulating factor (G-CSF), and interleukin-1 receptor antagonist (IL-1Ra), which suppress pro-inflammatory cytokines and promote mucosal tolerance (89, 90).

Macrophage polarization influences exosomal function and can be reciprocally influenced by exosomal cargo. Exosomes from healthy tissues typically promote anti-inflammatory M2 polarization, whereas those from diseased or inflamed tissues favor a pro-inflammatory M1 phenotype (91). Han et al. (92) found that small extracellular vesicles (sEVs) derived from Ptpn1-deficient macrophages enhanced intestinal epithelial barrier integrity and suppressed inflammation. These vesicles, rich in lactadherin, inhibited NF-κB activation in both macrophages and IECs, and favored M2 polarization. This aligns with earlier findings by Yang et al. (93), who showed that exosomes from M2b-polarized macrophages alleviated DSS-induced colitis by expanding Tregs, increasing IL-4, and reducing pro-inflammatory cytokines. M2-derived exosomes also stimulated epithelial proliferation and recovery via miR-590-3p-mediated cytokine suppression (94), while miR-93-5p-enriched M2 exosomes inhibited NET formation, decreased intestinal inflammation, and promoted tissue repair (95).

Other regulatory RNAs also contribute to the reparative function of MEX. The MEG3, previously identified in exosomes (96), was detected in M2-derived EVs. When transferred into inflamed colonic epithelial cells, it increased cell viability and reduced pro-inflammatory cytokine expression, further supporting MEX’s role in mucosal healing (97).

#### 3.2.4 T cell-derived exosomes

When acute inflammatory responses are insufficient, adaptive immune responses become necessary. In this context, T cell-derived extracellular vesicles (TEX) serve as important mediators of communication between T cells and other immune cells (98). Among these, Treg-derived exosomes are especially critical to maintaining immune homeostasis.

Treg secretion of enzymatically active, immunosuppressive exosomes in response to T cell receptor stimulation was demonstrated by Smyth et al. (99) and confirmed by Okoye et al. (100). Other suppressive T cell subsets, such as CD8^+^CD25^+^Foxp3^+^ cells, also release exosomes capable of inhibiting CD8^+^ T cell activity (101, 102). Similarly, thymic cell-derived exosome-like particles (ELPs) promote immune tolerance by supporting the generation Foxp3^+^ Tregs, which can convert CD4^+^CD25⁻ T cells into functional Tregs capable of suppressing effector T cell proliferation both *in vitro* and *in vivo*. This effect was partially mediated by TGF-β signaling, as neutralization of this cytokine impaired Treg induction (103). Clinical studies further support the immunosuppressive potential of TEX, as exosomes isolated from healthy individuals were more effective at inhibiting conventional CD4^+^ T cell proliferation (104).

A key mechanism of Treg-mediated regulation of immune response is the delivery of exosomal miRNAs to target cells. Activated Tregs release exosomes enriched with immunoregulatory miRNAs, including let-7d, miR-155, miR-150, and miR-142-3p, which are essential for their suppressive function (100, 101, 105). These exosomes may be taken up by Th1 cells, DCs, and B cells, reprogramming their functional phenotype. For instance, Treg-derived exosomes transfer miR-150-5p and miR-142-3p to DCs, inducing a tolerogenic phenotype characterized by increased IL-10 and reduced IL-6 production in response to LPS stimulation. The central role of miRNA cargo is underscored by the finding that Tregs lacking Dicer (essential for miRNA biogenesis) or Rab27a/b (required for exosome release) fail to suppress Th1 activity (100).

TEX also contributes to immune cell polarization. Treg-derived exosomes promote macrophage polarization toward M2 phenotype, elevating IL-10, IL-4, and IL-13 levels while reducing pro-inflammatory cytokine secretion (106). In line with this, Okoye et al. (100) demonstrated that delivery of Treg-derived exosomal Let-7d, to Th1 cells reduced Th1 proliferation and IFN−γ production. Collectively, these findings establish T cell-derived exosomes as key regulators of immune tolerance, acting through antigen-specific signaling, cytokine delivery, and miRNA-mediated reprogramming of immune cells.

#### 3.2.5 Others

In addition to exosomes derived from mast cells, macrophages, dendritic cells, and T cells, other immune cell populations, such as natural killer (NK) cells, B cells, neutrophils, and eosinophils release exosomes with emerging roles in gastrointestinal immunity and homeostasis.

NK cell-derived exosomes participate in both innate and adaptive immunity. They stimulate monocytes and T cells by upregulating HLA-DR and CD80/86 on monocytes and inducing CD25 on T cells, even in the presence of cytokines such as IL-10 and TGF-β, thereby demonstrating sustained immune activation under immunosuppressive conditions (107). In addition to their immunostimulatory properties, NK exosomes carry cytotoxic proteins such as perforin and granzymes, enabling the direct elimination of infected or transformed cells (107, 108). They also deliver immunomodulatory cytokines and regulatory microRNAs, potentially reinforcing mucosal defense while preserving immune balance.

B cell-derived exosomes similarly contribute to adaptive immunity. These vesicles express MHC class II molecules, co-stimulatory proteins such as CD80 and CD86, and antigen-peptide complexes, enabling them to function in antigen presentation and T cell activation (109). Recent work suggests that B cell-derived exosomes may also promote mucosal tolerance and contribute to the maintenance of gut immune equilibrium (110).

Neutrophil-derived exosomes are increasingly recognized for their dual role in gastrointestinal inflammation. During active inflammation, they can disrupt epithelial adhesion and contribute to barrier dysfunction, as observed in inflammatory bowel disease (IBD) models (111). Conversely, they may also participate in tissue repair, thus contributing to the restoration of intestinal homeostasis (112).

Collectively, these studies highlight immune cell-derived exosomes as significant regulators of gut homeostasis (Summarized in **Table 2**). Through integrated crosstalk, these exosomes shape immune polarization and tolerance, allowing immune cells to rapidly adapt to tissue and microbial-derived cues. Thus, balancing cytotoxic defense and antigen presentation with anti-inflammatory and pro-resolving responses. Dysregulation of these exosome-mediated pathways may therefore contribute to chronic intestinal inflammation and impaired mucosal healing.

**Table 2:** Immunomodulatory roles of immune cell-derived exosomes in gut homeostasis.

Cell of origin	Key cargo/mechanism	Biological function	Effect on gut physiology	Selected refs
Mast Cells (MCs)	OX40L	Drives Th2 cell differentiation.	Shapes adaptive immune responses.	(73)
	lncRNA NEAT1	Sponges miR-211-5p, upregulating GDNF.	Protects epithelial barrier integrity.	(74)
	mRNAs, miRNAs	Allows functional transfer between mast cells.	Facilitates indirect intercellular communication.	(12)
Dendritic Cells (DCs)	MHC II, ICAM-1 (from mature DCs)	Direct priming and activation of naïve T cells.	Initiates robust antigen-specific effector T cell responses.	(79, 81)
	miRNAs (from both mature and immature DCs)	Fuses with recipient DCs, delivering regulatory miRNAs.	Enables DC-DC communication and post-transcriptional regulation.	(82)
	TLR ligands	Cross-presented to “bystander” DCs, promoting their maturation.	Enhances sentinel function and NK cell activation.	(83)
	MHC II^+^, IL-10, TGF-β (from immature/tolerogenic DCs)	Suppresses alloreactive T cells; promotes Treg expansion.	Induces immune tolerance and dampens excessive activation.	(84–86)
Macrophages	Pro-inflammatory cytokines (TNF-α, IL-1β, IL-6)	Activates recipient cells; primes for immune challenges.	Contributes to immune activation (typically M1 phenotype).	(89, 90)
	Anti-inflammatory factors (TGF-β, FGF, IL-10, IL-1Ra, CCL1)	Suppresses pro-inflammatory cytokines.	Promotes resolution of inflammation and mucosal tolerance (typically M2 phenotype).	(89, 90)
	miR-590-3p, Lactadherin	Suppresses cytokines; inhibits NF-κB.	Stimulates epithelial proliferation, recovery, and suppresses inflammation.	(92, 94, 95)
	lncRNA MEG3	Increases cell viability, reduces pro-inflammatory cytokines in IECs.	Supports mucosal healing.	(97)
T Cells (Tregs)	CD73	mediators of peripheral tolerance	CD73-mediated generation of immunosuppressive adenosine.	(99)
	miRNAs (e.g., Let-7d, miR-150)	Treg-derived exosomes suppress Th1 cell proliferation, cytokine production	Essential for Treg suppressive function; via exosomal miRNA transfer.	(100, 101)
	miRNAs (miR-150-5p, miR-142-3p)	Treg-derived exosomes are taken up by DCs, inducing a tolerogenic phenotype.	Reprograms DCs to sustain a tolerogenic environment.	(105)
Others (NK, B, Neutrophils)	Perforin, Granzymes	Direct cytotoxic elimination of target cells.	Reinforces mucosal defense against infection/transformation.	(107, 108)
	MHC II, co-stimulatory proteins	Antigen presentation and T cell activation.	Contributes to adaptive immunity.	(109)
	(Neutrophil Exosomes)	Dual role: can disrupt epithelial adhesion or participate in repair.	Context-dependent role in inflammation and restoration of homeostasis.	(111, 112)

APC, antigen-presenting cell; DC, dendritic cell; DSS, dextran sulfate sodium; GDNF, glial cell line-derived neurotrophic factor; IEC, intestinal epithelial cell; lncRNA, long non-coding RNA; MC, mast cell; MHC, major histocompatibility complex; miRNA, microRNA; NET, neutrophil extracellular trap; NK, natural killer; Treg, regulatory T cell.

### 3.3 Bacteria-derived exosome-like vesicles in gut communication

The gut microbiota, comprising trillions of microorganisms, plays a fundamental role in shaping host immunity and maintaining gastrointestinal homeostasis (113). Through sustained co-evolution, the host and its microbiota have developed a mutualistic relationship where gut microbes educate and regulate the mucosal immune system, while the gut-associated immune system maintains tolerance to commensals. Gastrointestinal homeostasis therefore depends on tightly coordinated communication between gut microbiota, IECs, and the immune system (114, 115). In addition to host-derived extracellular vesicles, vesicles released by gut microbes act as vehicles for this inter-kingdom communication.

Bacteria-derived extracellular vesicles (BEVs), referred to as membrane vesicles (MVs) when secreted by Gram-positive bacteria and outer membrane vesicles (OMVs) when secreted by Gram-negative bacteria, are produced by both commensal and pathogenic microbes. These vesicles carry a variety of proteins, lipids, nucleic acids, and other immunomodulatory molecules (116, 117). BEVs deliver microbial antigens and microbe-associated molecular patterns (MAMPs) that are recognized by host pattern recognition receptors, including Toll-like receptors and NOD-like receptors. This recognition initiates downstream signaling cascades involving NF-κB, MAPKs, and IRFs, which regulate inflammatory gene expression and cytokine production (116–118). Notable examples include the delivery of immunomodulatory polysaccharide A (PSA) from *Bacteroides fragilis* OMVs and lipoteichoic acid (LTA) from Gram-positive *Lacticaseibacillus rhamnosus* JB-1 MVs, both of which promote TLR2 signaling and subsequent immune response (119, 120).

Beyond indirect activation via IECs, BEVs may traverse the intestinal epithelium and interact directly with immune cells in the lamina propria. This interaction can induce anti-inflammatory cytokine production, suppress effector T cell responses, prime the immune system, and promote tolerance and intestinal homeostasis (121). For instance, MVs from the commensal *Lactobacillus rhamnosus* JB-1 cross the intestinal epithelium and reach Peyer’s patches, where they induce tolerogenic DC phenotype and promote the expansion of IL-10-secreting Tregs (122). Similarly, OMVs from *Akkermansia muciniphila* contribute to gastrointestinal homeostasis by entering Peyer’s patches, activating B cells and DC, and triggering mucosal immunoglobulin A production. Likewise, MVs from *Bifidobacterium bifidum* LMG13195 stimulate the differentiation of tolerogenic DC and Tregs (123). Comparable immunomodulatory effects have been reported with the Gram-negative *Escherichia coli* Nissle 1917 (EcN), whose OMVs are readily internalized by macrophages, enhancing antimicrobial activity and promoting IL-10-skewed cytokine production (124). These effects appear to be strain-specific. BEVs from EcN and related commensal *E. coli* strains promote DC maturation and condition downstream T cell responses toward Th1, Th2, or Treg phenotypes, reflecting strain-specific vesicle-mediated immune education and underscoring the gut microbiota’s capacity to induce balanced immune responses (125, 126)

In addition to immune regulation, BEVs support epithelial barrier integrity, an essential component of gut homeostasis. BEVs from EcN and ECOR63 maintain epithelial integrity by upregulating TJ proteins such as occludin and ZO-1. In epithelial monolayers infected with enteropathogenic *E. coli* (EPEC), these OMVs preserve cytoskeletal organization and prevent barrier breakdown (127, 128). OMVs from EcN and ECOR12 also stimulate IL-22 and human β-defensin-2 expression in human colonic explants, enhancing mucosal defense through goblet and Paneth cell activation (121, 129). OMVs from *Akkermansia muciniphila* further support intestinal barrier function by entering epithelial cells and increasing the expression of occludin, claudin-4, and ZO-2, as well as promoting mucus production (130, 131).

BEVs can also reach systemic circulation under homeostatic conditions. For example, OMVs from *Bacteroides thetaiotaomicron* migrate paracellularly and have been detected in extra-intestinal tissues, suggesting a role in long-range host communication (132). BEVs have even been found in the placenta and amniotic fluid, where they may contribute to the development of immune tolerance before birth and prime the fetal immune system for postnatal microbial exposure (133).

In addition to host-microbe communication, BEVs mediate interbacterial interactions and competition. Early studies by Li et al. (134) demonstrated that OMVs from various Gram-negative species possess broad-spectrum bactericidal activity through peptidoglycan-degrading enzymes, which can kill competing bacteria by degrading their cell walls. More recent work by Dean et al. (135) showed that MVs from *Lactobacillus acidophilus* deliver bacteriocin peptides to suppress opportunistic pathogens, highlighting BEVs as potential interbacterial weapons. On the other hand, OMVs from *Akkermansia muciniphila* can fuse with and selectively promote the growth of beneficial gut commensals (131). BEVs from EcN may also help restore intestinal homeostasis by reducing the uptake of pro-inflammatory bacterial peptides through the normalization of dysregulated colonic peptide transport (136).

In sum, BEVs participate in maintaining gastrointestinal homeostasis, acting through immune education, tolerance induction, barrier reinforcement, microbial competition, and inter-kingdom communication (Summarized in **Table 3**).

**Table 3:** Roles of microbiota-derived extracellular vesicles in intestinal homeostasis.

Function	Vesicle source & type	Key mechanistic insights & effects	Outcome	Refs
Immune Tolerance & Regulation	Bacteroides fragilis(OMV)	Delivery of PSA to promote TLR2 signaling, leading to anti-inflammatory responses and Treg differentiation.	Suppression of excessive inflammation, maintenance of immune homeostasis.	(119)
	Lacticaseibacillusrhamnosus JB-1 (MV)	Carry LTA., induces tolerogenic DCs and IL-10-secreting Tregs.	Promotion of mucosal tolerance.	(120, 122)
	Bifidobacterium bifidum LMG13195 (MV)	Directly stimulates the differentiation of tolerogenic DCs and Tregs.	Induction of regulatory immune responses.	(123)
	Escherichia coli Nissle1917 (OMV)	Internalized by macrophages, promoting IL-10, antimicrobial activity. Conditions DCs to steer T-cell responses (Th1/Th2/Treg).	Strain-specific immune education and balanced immune priming.	(124, 125, 126)
Barrier Fortification	Escherichia coli Nissle1917, ECOR63 (OMV)	Upregulates TJ proteins (occludin, ZO-1). Prevents cytoskeletal disruption caused by enteropathogenic E. coli.	Preservation of intestinal barrier integrity and function.	(127, 128)
	Akkermansiamuciniphila (OMV)	Enters epithelial cells, increases occludin, ZO-1 and ZO-2 expression. Promotes mucus production.	Enhanced physical and chemical barrier defense.	(130, 131)
	Escherichia coli Nissle1917, ECOR12 (OMV)	Stimulates IL-22 and human β-defensin-2 expression.	Enhanced mucosal defense and innate immunity.	(121, 129)
Systemic Signaling	Bacteroides thetaiotaomicron (OMV)	Migrates paracellularly across epithelium; detected in extra-intestinal tissues.	Potential for long-range, systemic host communication.	(132)
	Various Commensals(BEVs)	Found in placenta and amniotic fluid.	May contribute to fetal immune priming; prenataltolerance development.	(133)
Microbial Competition	Lactobacillus acidophilus (MV)	Acts as a vehicle for targeted delivery of bacteriocin peptides to competingbacteria.	Selective suppression of opportunistic pathogens.	(135)
	Various Gram-negative species (OMV)	Deliver peptidoglycan-degrading enzymes with broad-spectrum bactericidal activity.	Killing of competing bacterial cells.	(134)
	Akkermansiamuciniphila (OMV)	Fuses with and up-regulates beneficial bacteria.	Selective promotion of symbiotic microbial communities.	(131)
HomeostasisRestoration	Escherichia coli Nissle1917 (OMV)	Delivers miRNA (e.g., miR-193a-3p) to reduce expression of dysregulated colonic peptide transporter (PepT1).	Limits uptake of pro-inflammatory peptides, amelioration of inflammation.	(137)

BEV, Bacteria-Derived Extracellular Vesicle; OMV, Outer Membrane Vesicle (Gram-negative bacteria); MV, Membrane Vesicle (Gram-positive bacteria); TJ, Tight Junction; PSA, Polysaccharide A; LTA, Lipoteichoic Acid; TLR2, Toll-Like Receptor 2; DC, Dendritic Cell; Treg, Regulatory T Cell; Th, T helper cell; ZO-1, Zonula Occludens-1; IL, Interleukin; Ig, Immunoglobulin.

## 4 Exosomes in IBD pathophysiology

Inflammatory bowel disease, which encompasses Crohn’s disease (CD) and ulcerative colitis (UC), is a remitting and relapsing condition characterized by chronic inflammation of the gastrointestinal tract (137, 138). While the precise etiology of IBD remains incompletely understood, disruption of mucosal immune homeostasis and abnormal interactions between the host immune system and gut microbiota, particularly in genetically susceptible individuals, are known to contribute to disease pathogenesis (139). In the chronically inflamed intestinal mucosa, exosome-mediated signaling is dysregulated, leading to epithelial barrier dysfunction, aberrant immune activation, gut microbiota dysbiosis, and the persistent inflammation typical of IBD (13, 17). This section discusses key aspects of IBD-related exosomal abnormalities and explores the molecular mechanisms by which these vesicles may drive disease progression (Summarized in **Table 4**).

**Table 4:** Mechanisms of exosome-mediated pathogenesis in IBD.

Pathogenic process	Source of exosomes/Key cargo	Mechanism of action	Resulting pathology	Refs
Amplifying Inflammation	CD14+ intestinal macrophages Cargo: Membrane-bound TNF	TNFR2/NF-κB signaling → Induces a metabolic shift to glycolysis.	Sustains a pool of activated immune cells and drives chronic inflammation in CD.	(155, 156)
		Protect CD4+ T Cells from Activation-induced Cell Death		
	M1 Macrophages Cargo: Pro-inflammatory mediators	Activates TLR4 signaling in recipient cells.	Exacerbates colitis progression and mucosal barrier injury.	(154)
	Inflamed intestinal tissue Cargo: IL-6, IL-8, TNF-α, IL-10	Promotes IL-8 secretion from epithelial cells and enhances macrophage migration.	Amplifies the inflammatory response.	(153)
	IECs/dsDNA (mtDNA, nDNA)	Internalized by macrophages → Activates the cGAS-STING pathway → Leads to pro-inflammatory cytokine production.	Aggravates inflammation, particularly in Crohn’s disease.	(51)
	Immune cells/lncRNA NEAT1	Stabilizes TNFRSF1B (TNFR2) mRNA → Activates NF-κB signaling → Increases IL-8 and MCP-1 secretion.	Promotes M1 macrophage polarization and chronic inflammation.	(157, 158)
Inflammasome Activation	Macrophages/Monocytes Cargo: IL-1β, Caspase-1, ASC, MHC-II	P2X7 receptor activation by extracellular ATP (high in IBD) triggers the release of exosomes containing inflammasome components and mature IL-1β.	Mechanism for bioactive IL-1β export, contributing to chronic inflammation.	(164, 165)
	IECs Cargo: IL-1β, GSDMD	GSDMD facilitates the sorting of IL-1β into exosomes (non-pyroptotic release). These sEVs also contain NEDD4, Caspase-8.	Provides an alternative, non-pyroptotic pathway for IL-1β release that is active in IBD	(169)
Lipid-mediated inflammation	Mast Cell line Cargo: Phospholipases, arachidonic acid, PGE_2_, COX enzymes	Arachidonic acid cascade	Amplification of prostaglandin-driven inflammation	(160)
Promoting Fibrosis	Mesenteric adipose tissue (MAT) Cargo: TINAGL1	TINAGL1 binds SMAD4 in colonic fibroblasts → Activates TGF-β/SMAD signaling → Upregulates α-SMA and Collagen.	Directly promotes the activation of fibroblasts, driving intestinal fibrosis.	(181)
	IECs Cargo: TGF-β1 mRNA	Activates fibroblasts, increasing α-SMA and collagen expression.	Initiates pro-fibrotic tissue responses and regeneration.	(59, 178)
Breaking Barrier Integrity	Serum/Colonic Tissue/miR-21	Colonic mucosa of the TNBS group showed severe inflammatory reactions	Aggravates TNBS-induced colitis injury	(182, 183)
	M1 Macrophages/miR-21a-5p	Downregulates E-cadherin expression in IECs. Also activates ILC2s and increases Th2 cytokinerelease.	Disrupts adherens junctions and amplifies inflammation, compromising the barrier.	(190)
	Macrophages/miR-223	Downregulates TMIGD1 (a barrier-protective protein) in IECs.	Impairs mucosal integrity and repair.	(191)
	Mast cells/miR-223	Directly targets the 3’ UTR of CLDN8 mRNA, suppressing its translation.	Loss of tight junction integrity, barrier dysfunction	(172)
	Prdx3-deficient IECs Cargo: miR-1260b	Exosomal miR-1260b transfer activates p38 MAPK/NF-κB signaling and increases ROS production in recipient cells.	Damages tight junctions and exacerbates colitis.	(170)
	Serum, IECs/lncRNA NEAT1	Decreased expression of tight junction proteins (ZO-1, occludin, claudin-5), M1-type polarization	Exacerbates epithelial damage and inflammation. epithelial permeability,	(195)
Microbiota-Driven Mechanisms	AIEC-infected IECs Cargo: (general)	AIEC infection triggers host exosome release that activates innate immune responses (TNF-α) but also enhances AIEC intracellular replication.	Creates a vicious cycle of inflammation and bacterial survival.	(200)
	AIEC-infected IECs Cargo: miR-30c, miR-130a	Suppress expression of autophagy genes (ATG5, ATG16L1) in recipient IECs.	Impairs autophagy-mediated bacterial clearance, facilitating intracellular AIEC persistence.	(202)
	AIEC-infected IECs (Suppression of let-7b)	Loss of exosomal let-7b removes its repression of TGFβR1 in macrophages → Enhances pro-fibrotic macrophage activity.	Exacerbates intestinal fibrosis in Crohn’s disease.	(201)
	*F. nucleatum*-infected IECs Cargo: miR-129-2-3p	Targets TIMELESS → Activates the ATM/ATR/p53 pathway → Induces epithelial senescence.	Induces a senescence-associated secretory phenotype (SASP), impairing barrier function.	(203)
	ETBF exposure (Suppression of miR-149-3p)	ETBF suppresses host exosomal miR-149-3p, loss of exosomal miR-149-3p promoted Th17 differentiation	IL-17A/IL-6/TNF-α upregulation, driving inflammation	(204)
	Enterobacteriaceae particles → Host IECs Cargo: S1P	Bacterial particles induce IECs to produce exosome-like vesicles enriched with Sphingosine-1-Phosphate (S1P).	S1P acts as a chemotactic factor to recruit and promote the proliferation of Th17 cells.	(205)

AIEC, Adherent-invasive Escherichia coli; ETBF, Enterotoxigenic Bacteroides fragilis; IEC, intestinal epithelial cell; S1P, Sphingosine-1-phosphate; TNFR2, TNF receptor 2; STING, Stimulator of interferon genes; GSDMD, Gasdermin D; ROS, reactive oxygen species; TGF-β, Transforming growth factor beta; α-SMA, alpha-smooth muscle actin; ILC2, type 2 innate lymphoid cell.

### 4.1 Quantitative and molecular changes in exosomes

Alterations in the quantity and molecular composition of exosomes have been documented in individuals with IBD, suggesting that these changes contribute to disease pathogenesis while reflecting molecular alterations associated with disease. Elevated levels of circulating exosomes have been reported in the serum, feces, and saliva from patients with active IBD (140, 141). Moreover, the small GTPases RAB27A and RAB27B, which are involved in exosome secretion, are increased in immune cells from the colonic mucosa of patients with active UC (43, 142).

Compositional changes in both nucleic acids and proteins have also been reported. Most circulating miRNAs in serum and saliva are exosome-bound, and may become dysregulated in IBD (143, 144). For example, let−7b−5p was among differentially enriched miRNAs in CD-derived serum exosomes (145), the circular RNA Circ_0001187 was highly expressed in the serum exosomes of UC patients (Ouyang et al., 2022), while exosomal double-stranded DNA (dsDNA), including mitochondrial (mtDNA) and nuclear (nDNA) DNA, was elevated in the plasma of both experimental and human colitis (146).

Proteins involved in immunoregulation and inflammation are also altered in IBD exosomes. Pregnancy zone protein (PZP), an immunosuppressive factor, and the pro-resolving protein ANXA1 are significantly elevated in exosomes from IBD patients and colitic mice (55, 147). Proteasome subunit alpha type 7 (PSMA7), associated with proteasome activity and inflammation, is markedly increased in salivary exosomes from both Crohn’s and UC patients (140). More recently, Yang et al. (141) demonstrated that salivary exosomes from individuals with active IBD worsened experimental colitis, supporting the idea that these compositional changes are not merely byproducts of disease but may actively contribute to its progression.

Beyond systemic fluids, exosomes derived directly from inflamed intestinal tissues contain distinct molecular cargo. Elevated levels of exosomal dsDNA were detected in colon lavage samples from colitic mice and patients with active CD (146). Additionally, extracellular vesicles (mean size 146 ± 0.5 nm) from inflamed sites differed markedly in RNA content and exerted pro-inflammatory effects on epithelial and immune cells (147, 148). Collectively, these findings indicate that quantitative and compositional changes in exosomes not only reflect IBD activity but that these exosomes may actively participate in disease pathogenesis, highlighting their potential as non-invasive biomarkers for monitoring disease.

### 4.2 Exosomes as amplifiers of mucosal inflammation

**Figure 3:**
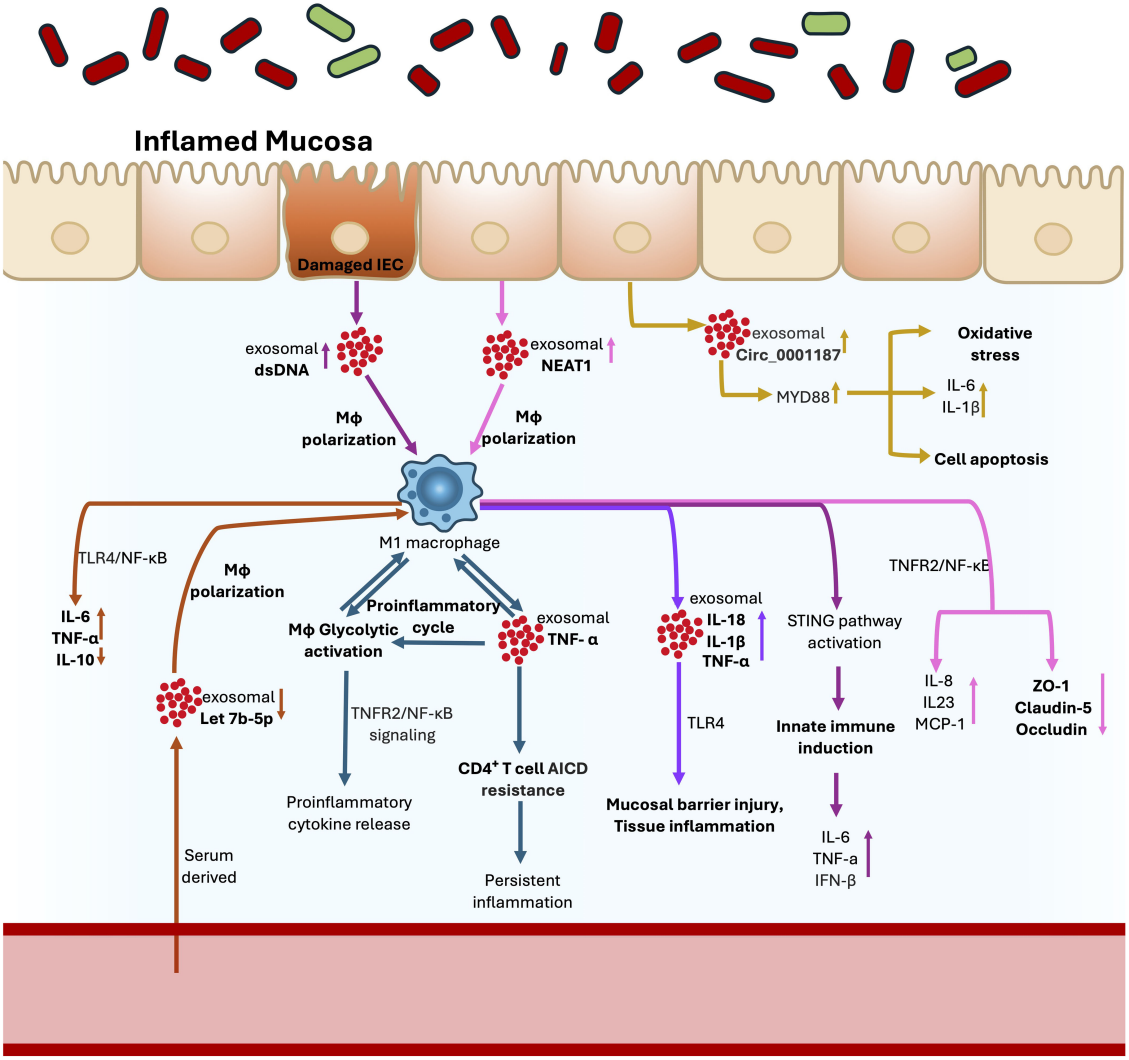
Exosomes as drivers of chronic inflammation in IBD. Exosomes act as critical mediators sustaining mucosal inflammation in IBD through multiple interconnected mechanisms. M1-polarized macrophages release exosomes enriched in IL-1β, IL-18, and TNF-α, which aberrantly activate TLR4 signaling, disrupt epithelial integrity, and promote inflammation. CD14^+^ intestinal macrophages secrete TNF-bearing exosomes that engage TNFR2, driving NF-κB–dependent glycolytic reprogramming, amplifying cytokine release, and creating a self-sustaining inflammatory loop. These vesicles also protect CD4^+^ T cells from activation-induced cell death (AICD), maintaining pools of activated effector T cells that fuel chronic inflammation. Damaged IECs contribute by releasing exosomes carrying dsDNA, which activate STING signaling in macrophages, aggravating inflammatory cytokine production (IL-6, TNF-α, IFN-β) and further damaging the epithelium. Additionally, dysregulated exosomal non-coding RNAs drive immune dysregulation: lncRNA NEAT1 promotes M1 polarization via NF-κB activation, while circRNA Circ_0001187 exacerbates epithelial inflammation by sponging miR-1236-3p, upregulating MYD88, and enhancing NF-κB–dependent signaling. Collectively, these exosome-driven pathways amplify immune activation, impair barrier integrity, and perpetuate chronic mucosal inflammation in Crohn’s disease and ulcerative colitis.

In IBD, persistent activation of pro-inflammatory pathways and immune-cell recruitment drive chronic inflammation. Exosomes released by activated immune cells are key to sustaining this inflammatory state, carrying immunomodulatory molecules that aberrantly stimulate both innate and adaptive immune responses (**Figure 3**). For instance, exosomes from M1-polarized macrophages were shown to exacerbate colitis through aberrant activation of TLR4 signaling (149). Adding to this, CD14^+^ intestinal macrophages have been identified as a major source of pro-inflammatory exosomes in IBD. Liu et al. (150) showed that these intestinal macrophage-derived vesicles, enriched in membrane-bound TNF, protected CD4^+^ T cells from activation-induced cell death, thereby maintaining pools of activated T cells that drive chronic inflammation. Expanding on this, Zeng et al. (151) showed that the same TNF-bearing exosomes reprogrammed macrophage metabolism to sustain inflammation in CD. Mechanistically, exosomal TNF molecules engaged the TNFR2 receptor on intestinal macrophages, triggering glycolytic activation via NF-κB signaling in both an autocrine and paracrine manner. This glycolytic shift increased production of pro-inflammatory cytokines, creating a self-amplifying metabolic–immune feedback loop that perpetuates inflammation in CD.

Dysregulated expression of functional exosomal biomolecules also contributes to persistent mucosal inflammation (144). The lncRNA NEAT1, which is elevated in IBD, promoted exosome-mediated M1 polarization by stabilizing TNFRSF1B mRNA and activating NF-κB signaling, leading to increased IL-8, IL-23 and MCP-1 secretion (152, 153). Likewise, serum derived exosomal let-7b-5p activated macrophages through TLR4/NF-κB signaling (145), while circRNA Circ_0001187 promoted epithelial inflammation by sponging miR-1236-3p, upregulating MYD88, and driving NF-κB–dependent pathways (154). Exosomes released from damaged IECs also transported dsDNA to macrophages, triggering STING activation and aggravating inflammation in Crohn’s disease (146). In parallel, extracellular vesicles (~146 nm in diameter) derived from inflamed intestinal tissues contained elevated levels of IL-6, IL-8, IL-10, and TNF-α, which promoted IL-8 secretion and enhanced macrophage migration (148). In addition to nucleic acids and cytokines, exosomes may perpetuate inflammation by transporting bioactive lipids and enzymes involved in pro-inflammatory signaling. For example, exosomes from the immune cell line RBL-2H3 were found to contain phospholipases, arachidonic acid, prostaglandin E_2_, and cyclooxygenase enzymes, all key components of the arachidonic acid pathway implicated in IBD (155).

#### 4.2.1 Inflammasomes activation

Activation of inflammasomes, cytosolic complexes that detect pathogen- or damage-associated molecular patterns, contributes to chronic intestinal inflammation (156). Among others, the NOD-like receptor family pyrin domain containing 3 (NLRP3) inflammasome, which can be triggered by mitochondrial dysfunction, reactive oxygen species (ROS), lysosomal damage, or ion flux, is very relevant to IBD, as it drives the maturation of IL-1β and IL-18 and induces pyroptosis via gasdermin D (GSDMD) (157, 158). Exosomes facilitate this process by transporting relevant cytokines and inflammasome components. Qu et al. (159), while investigating the rapid export of IL-1β from monocytes/macrophages upon P2X7 receptor (P2X7R) activation, found that activation of the P2X7R by extracellular ATP led to the release of exosomes containing IL-1β, caspase-1, and inflammasome components. Subsequent work from the same group showed these exosomes were MHC-II positive and released from macrophages and dendritic cells (160). Since P2X7 is overexpressed and persistently activated by high extracellular ATP in IBD, this mechanism likely contributes to chronic exosome-driven inflammasome activity (160–162). Additional studies have linked GSDMD to non-pyroptotic exosome-mediated inflammasome regulation. In IECs, GSDMD facilitated the release of IL-1β–containing sEVs by tagging pro–IL-1β for sorting into secretory vesicles. These sEVs co-packaged GSDMD, NEDD4, caspase-8, and IL-1β, while expressing canonical exosomal markers such as CD63 and ALIX. Notably, GSDMD expression was elevated in both IBD patients and colitic mice, implicating this exosomal route in disease pathogenesis (163). Mitochondrial oxidative stress may further trigger NLRP3 activation via exosomal signaling. Knockdown of the mitochondrial antioxidant peroxiredoxin 3 in colonic epithelial cells worsened colitis by upregulating release of exosomal miR-1260b, which exacerbated inflammation (164). On the other hand, miR-223, enriched in MC-EXOs and upregulated in IBD tissues and preclinical colitis models, was shown to negatively regulate NLRP3 activation (165–167). Mice lacking miR-223 exhibited heightened susceptibility to experimental colitis, with increased expression of NLRP3 inflammasome components, elevated IL-1β, and greater monocyte infiltration (168). These studies suggest that miR-223 may be a regulatory mechanism to curb excessive inflammasome activation, although other studies have reported pro-inflammatory effects for this miRNA (165, 166, 169).

In summary, exosomes amplify mucosal inflammation in IBD through multiple complementary mechanisms, including the delivery of pro-inflammatory RNAs and proteins, metabolic reprogramming of immune cells, transport of bioactive lipids, and modulation of inflammasome activity.

### 4.3 Exosomes promote fibrosis in IBD

Exosomal signaling contributes to tissue remodeling and fibrosis, a common complication of IBD. Intestinal fibrosis is primarily driven by soluble mediators including growth factors (170). Among these, dysregulation of TGF-β signaling, a key regulator of immune homeostasis, is an important feature of IBD. Although TGF-β levels are elevated in inflamed tissues, its downstream signaling pathways are often impaired. As a result, TGF-β fails to exert sufficient immunosuppressive effects during chronic inflammation and may instead promote the fibrotic phenotype characteristic of IBD (171). Exosomes contribute to this process by transporting TGF-β and fibrosis-related regulatory molecules. For example, in response to injury, IECs themselves release TGF-β-containing exosomes that activate fibroblasts and initiate pro-fibrotic tissue regenerative processes (59). Moreover, miR-200b, often packaged in exosomes, inhibits TGF-β–driven pro-fibrotic responses but is significantly reduced in the intestinal mucosa of IBD patients (172, 173). A recent study also highlighted the contribution of mesenteric adipose tissue (MAT)–derived exosomes in promoting intestinal fibrosis in CD via the TINAGL1/SMAD4/TGF-β signaling axis (173). Using a dinitrobenzene sulfonic acid (DNBS)-induced chronic colitis mouse model to mimic fibrotic CD, these authors showed that exosomes from fibrotic MAT were enriched in TINAGL1, which binds directly to SMAD4 and activates TGF-β signaling. *In vitro*, these exosomes promoted human colonic fibroblast activation, increasing collagen and α−SMA expression. Furthermore, recombinant TINAGL1 treatment *in vivo* worsened intestinal fibrosis, underscoring the pathogenic role of this exosome-associated signaling axis in fibrostenotic IBD (173).

### 4.4 Exosome-mediated breakdown of epithelial barrier integrity

Epithelial barrier dysfunction is a central feature of IBD, and growing evidence implicates exosomes and their RNA cargo in driving this process. Among these, miR-21 is dysregulated in both IBD tissues and exosomes, where it targets genes essential for TJ maintenance (174). Elevated miR-21 levels have been detected in colonic biopsies and serum of UC patients, with localization specifically to IECs (175). The neuropeptide Substance P, which is elevated in IBD and implicated in both pro- (176) and anti-inflammatory (177) activities, enhanced exosome production and selectively enriched miR-21 in the exosomal cargo of colonic epithelial cells (178). Functional studies indicate that miR-21 disrupts epithelial barrier function by targeting RhoB and CDC42, key regulators of junctional integrity (175, 179). Li et al. (180) further identified a pro-inflammatory IL-9/miR-21/CLDN8 axis in CD, in which IL-9 upregulation increased miR-21, suppressing CLDN8 expression, thus resulting in barrier disruption. Interestingly, miR-21’s role in immune and barrier regulation may vary across inflammatory settings; its deletion worsened inflammation in TNBS and T-cell transfer colitis models but attenuated disease severity in DSS-induced colitis, suggesting a complex, model-specific function (181).

Immune cell-derived exosomes also impair epithelial integrity. miR-21a-5p, elevated in M1 macrophage-derived exosomes from UC patients and DSS-treated mice, disrupted the epithelial barrier by downregulating E-cadherin, activating ILC2s, and increasing Th2 cytokine release, ultimately amplifying inflammation (182). Similarly, exosomes from LPS-stimulated macrophages carry miR-223, which downregulates TMIGD1, a barrier-protective protein, further impairing mucosal integrity (183). Mast cell-derived exosomes, also enriched with miR-223, were taken up by epithelial cells where they reduced the expression of ZO-1, occludin, and claudin-8, increasing epithelial permeability (166). Note that an earlier study had established that miR-223 directly targets CLDN8 mRNA, thus identifying a IL-23/miR-223/CLDN8 pathway linking the IL-23/Th17 immune axis to barrier disruption (184). These findings are particularly significant since immune cell infiltration is markedly increased in the intestinal mucosa of IBD patients.

Other exosomal RNA similarly contribute to barrier breakdown (11, 185). Elevated exosomal miR-1260b activated p38 MAPK/NF-κB signaling, increasing ROS production, damaging TJs, and exacerbating colitis (164) Conversely, silencing of exosomal lncRNA NEAT1 improved barrier integrity by upregulating ZO-1, Occludin, and Claudin-5 (152, 185).

Lastly, exosomal lipids may impact epithelial barrier regulation. Exosomal lipid rafts play a fundamental role in exosome biogenesis and cargo selection (186). Cholesterol, a major raft component, is essential for epithelial barrier function as it supports tight junction (TJ) formation by organizing proteins such as occludin and claudins within these microdomains (187, 188). Its depletion disrupted this organization, resulting in TJ disassembly and increased epithelial permeability. Indeed, loss of cholesterol-rich rafts occurs early in both murine and human IBD, suggesting that lipid raft disruption may be an initiating event in barrier breakdown, rather than a consequence of established inflammation (189). Given these findings, it is plausible that disruption of exosomal lipid raft components, coupled with the dysregulated exosome release observed in IBD, contributes to barrier dysfunction. While direct evidence linking exosomal lipid rafts to barrier regulation in IBD is limited, these vesicles represent a potentially underexplored mechanism linking chronic inflammation to epithelial barrier failure.

### 4.5 Microbiota-exosome crosstalk in IBD pathogenesis

**Figure 4:**
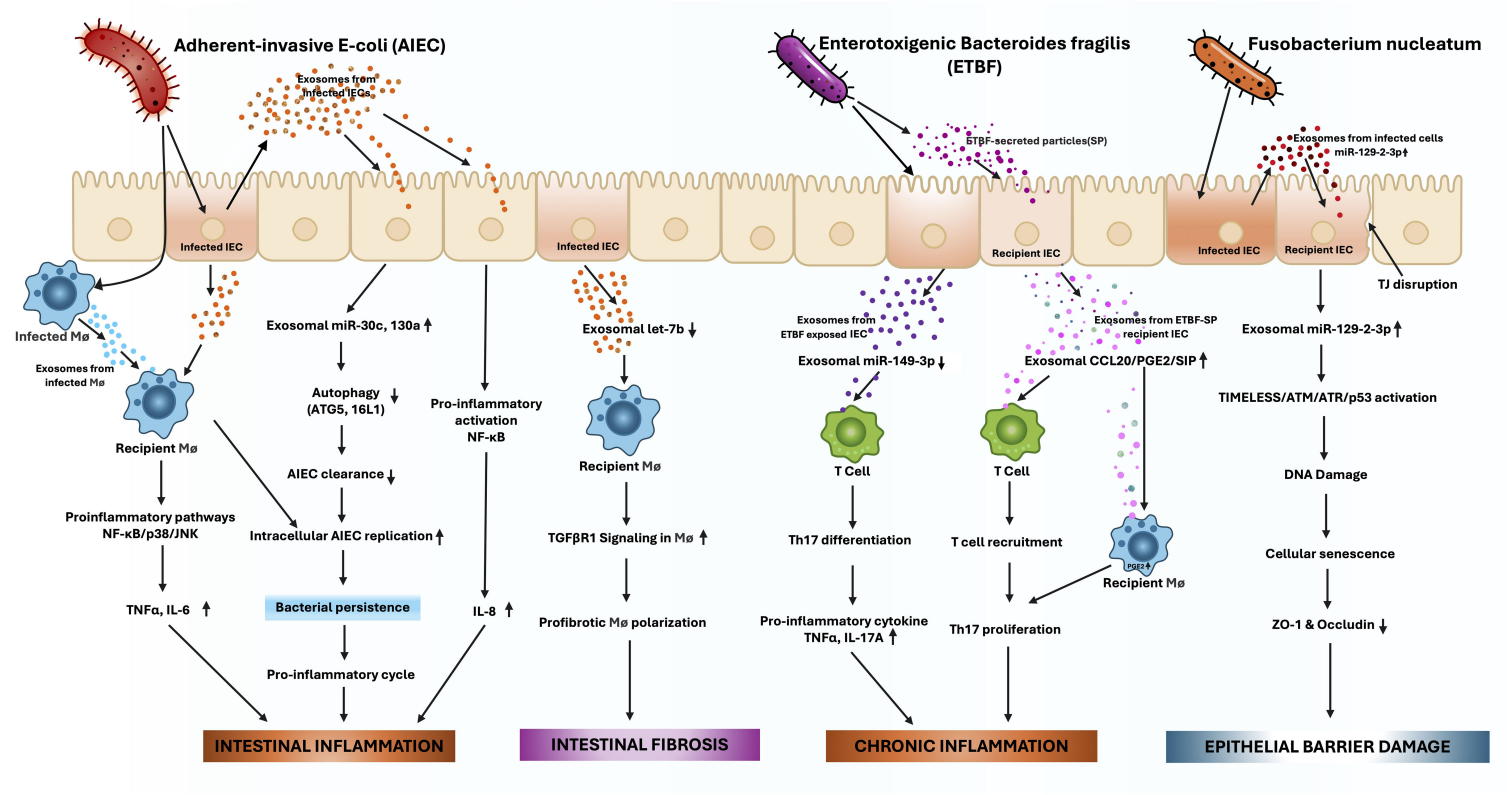
Pathobiont-driven exosome dysregulation in IBD pathogenesis. Pathobionts alter intestinal epithelial cell (IEC)-derived exosomal cargo, promoting inflammation, fibrosis, and barrier dysfunction. Adherent-invasive Escherichia coli (AIEC)infection induces IEC exosomes enriched with miR-30c and miR-130a, which suppress autophagy-related proteins ATG5 and ATG16L1, impairing bacterial clearance and sustaining intracellular replication. These exosomes activate proinflammatory macrophage pathways (NF-κB, p38, JNK), leading to increased TNFα and IL-6, bacterial persistence, and an inflammatory cycle. AIEC also downregulates exosomal let-7b, enhancing TGFβR1 signaling in macrophages, driving profibrotic polarization and intestinal fibrosis. Enterotoxigenic Bacteroides fragilis (ETBF) exposure decreases exosomal miR-149-3p from epithelial cells, enhancing Th17 differentiation and contributing to inflammation and tumorigenesis. In parallel, ETBF-secreted particles (ETBF-SP) stimulate IEC exosomes containing sphingosine-1-phosphate (S1P), CCL20, and PGE2, which promote Th17 recruitment and amplify mucosal inflammation. Fusobacterium nucleatum infection triggers exosomal miR-129-2-3p release, which targets the TIMELESS/ATM/ATR/p53 pathway, leading to DNA damage, cellular senescence, and disruption of tight junction proteins ZO-1 and occludin, thereby promoting epithelial barrier damage.

Gut microbiota dysbiosis is now a well-established hallmark of IBD. Altered exosomal signaling shapes microbial composition and contributes directly to disease pathogenesis (**Figure 4**). Carrière et al. (190) showed that exosomes released from epithelial and immune cells infected with adherent-invasive *Escherichia coli* (AIEC), a pathobiont linked to CD, activate host innate immunity. Xu et al. (191) further revealed that AIEC infection promotes intestinal fibrosis by suppressing epithelial release of exosomal let-7b, a microRNA that normally limits pro-fibrotic macrophage activity by targeting TGFβR1. Loss of let-7b therefore exacerbated intestinal fibrosis in CD. Similarly, AIEC-infected epithelial cells secreted exosomes enriched with miR-30c and miR-130a, which suppressed autophagy-related genes ATG5 and ATG16L1 in recipient cells. Impaired autophagy diminished bacterial clearance, facilitating AIEC persistence (192).

Other enteropathogens exert comparable effects. *Fusobacterium nucleatum* (Fn), elevated in the colonic tissue of UC patients, modifies the microRNA cargo of IEC-derived exosomes. Wei et al. (193) found that Fn-infected IEC exosomes were enriched in miR-129-2-3p, which targets TIMELESS, a regulator of DNA repair and senescence. By activating the ATM/ATR/p53 pathway, these vesicles induced epithelial senescence, impaired barrier function, and worsened colitis. Likewise, enterotoxigenic *Bacteroides fragilis* suppressed exosomal miR-149-3p in colonic epithelial cells, driving Th17 cell differentiation and promoting intestinal inflammation (194).

Enteropathogenic bacteria also stimulated IECs to secrete exosomes containing sphingosine-1-phosphate, CCL20, and prostaglandin E2, which amplified inflammatory response by promoting Th17 recruitment and proliferation, contributing to the Treg/Th17 imbalance that sustains IBD-associated inflammation (85, 195).

In addition to pathogen-induced disruption, host-derived exosomal microRNAs may also regulate the gut microbiota. Host-derived fecal miRNAs (often packaged in exosomes), primarily from IECs and Hopx-positive cells, can enter gut bacteria, regulate bacterial gene expression, and influence microbial growth. Mice lacking epithelial miRNAs (Dicer1^ΔIEC) developed microbial dysbiosis and aggravated colitis, both of which were reversed by fecal miRNA transplantation from wild -type mice, demonstrating a direct role for fecal miRNAs in microbiota regulation and intestinal inflammation (196). Consistent with this, Casado-Bedmar et al. (197) found that fecal miRNAs such as miR-21 and let-7b not only altered microbiota composition but also promoted secretion of myeloperoxidase and antimicrobial peptides, fostering microbiota dysbiosis, barrier dysfunction, and colitis. Collectively, these studies position exosomes as bidirectional messengers in host-microbiota interaction in IBD. Host-derived exosomal cargo drives gut dysbiosis and exacerbates gut inflammation. In parallel, pathogen-derived signals reshape host exosomal content to modulate microbial colonization of the gut and drive bacterial persistence, thereby altering epithelial barrier function, mucosal immune signaling, and sustaining chronic inflammation. However, the functional roles of individual exosomal cargo remain context-dependent, and their translational potential is still at an early stage.

## 5 Therapeutic potential of exosomes in IBD

**Figure 5:**
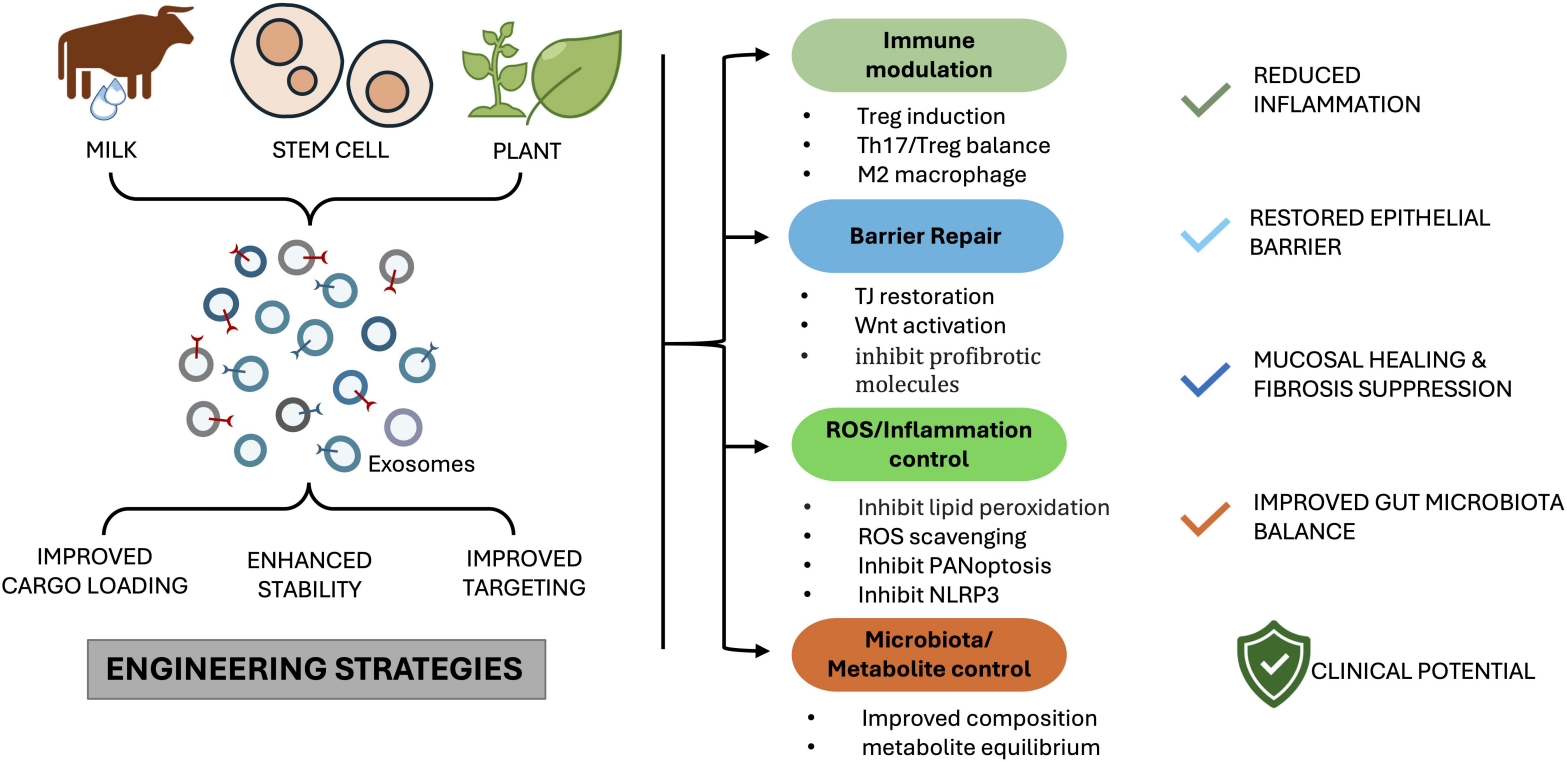
Therapeutic potential of exosomes in IBD. Exosomes from diverse sources, including milk, mesenchymal stem cells (MSCs), plants, and engineered sources, contribute to mucosal protection and repair. Their mechanisms include immune modulation through induction of regulatory T cells, promotion of M2 macrophage polarization, and restoration of Th17/Treg balance; regulation of programmed cell death pathways such as pyroptosis, ferroptosis, and apoptosis; reinforcement of epithelial barrier integrity and regeneration via Wnt/β-catenin signaling and fibrosis inhibition; modulation of gut microbiota composition and metabolite production; and suppression of oxidative stress and inflammation through NLRP3 inflammasome inhibition, reactive oxygen species scavenging, and NF-κB suppression. Collectively, these effects reduce inflammation, restore barrier function, and promote mucosal healing, highlighting the translational promise of exosome-based therapies in IBD.

Exosomes are a promising therapeutic tool for IBD due to their intrinsic ability to carry bioactive cargo, their biocompatibility, and their relative stability in the gastrointestinal tract. Native vesicles from milk, stem cells, plants, and other sources have shown anti-inflammatory, barrier-restorative, and microbiota-modulating effects in preclinical models, while engineering strategies are enhancing their cargo loading, targeting specificity, and stability during transit (**Figure 5**) (198, 199).

Milk-derived exosomes from goat, cow, bovine, and human sources reduce inflammation, strengthen intestinal epithelial barrier function, and favorably modulate gut microbiota and metabolite profiles in colitis models (200, 201). These vesicles demonstrate significant bioavailability in target tissues, although therapeutic efficacy may require dose optimization or cargo enrichment (202).

Stem cell-derived exosomes from mesenchymal and perinatal sources exert potent immunoregulatory and regenerative effects. They promote Treg induction, drive M2 macrophage polarization, and deliver miRNAs that inhibit pyroptosis and ferroptosis in immune and epithelial cells (203–206). These vesicles also suppress NLRP3 inflammasome activation, restore Th17/Treg balance, promote epithelial repair through Wnt/β-catenin signaling, alleviate IBD-associated fibrosis via ERK inhibition, and suppress IEC apoptosis through modulation of histone acetylation (207–211). In another study, umbilical cord MSCs improved IBD-like symptoms, with greater therapeutic benefits when co-administered with MSC exosomes and mesalazine. Notably, exosomes alone lacked sufficient efficacy, suggesting they may be most effective in combination therapy (212). Early clinical data are also encouraging: in a phase I trial, MSC-derived exosomes for refractory perianal fistula was well tolerated, and showed significant therapeutic effect (213).

Plant-derived exosome-like nanoparticles (ELNs) from ginger, ginseng, and tea offer a scalable and edible option, naturally suited for oral delivery. ELNs can traverse the mucosal layer, and their lipid composition enhances stability and uptake, offering advantages over direct plant consumption. In colitis models, they support epithelial barrier resilience, anti-inflammatory pathways, and antimicrobial peptide expression (214–217).

Engineering strategies further extend the therapeutic potential of native exosomes. Current approaches have emphasized three main engineering goals: increasing cargo-loading efficiency, improving targeting to inflamed mucosa, and enhancing stability during gastrointestinal transit (198). Examples include milk exosomes loaded with anti-TNF siRNA for oral administration (218), hucMSC exosomes coated with poly(lactic-co-glycolic acid) (PLGA) to withstand the harsh environment of the gastrointestinal tract (219), and plant ELNs delivering CX5461 for macrophage-targeted therapy (217). Additional strategies that broaden therapeutic options include probiotic-conditioned IEC exosomes that modulate macrophage polarization (220), rectally delivered hydrogel-embedded miR-23a-3p-rich exosome releasing amniotic epithelial stem cells that suppress TNFR1–NF-κB signaling (221), as well as Treg-derived exosomes loaded with selenium and modified with a mitochondria-targeting peptide for site-specific activation in inflamed tissue (222). Similar recently developed novel exosome systems such as cerium oxide-loaded Treg exosomes ameliorated IBD by scavenging ROS and modulating inflammatory responses (223).

Overall, vesicle source, cargo engineering, and delivery format shape the therapeutic potential of exosome-based therapy for IBD. Optimizing these parameters may allow for tailored interventions that address specific clinical needs.

## 6 Conclusion and future perspective

Exosomes have emerged as important regulators of gastrointestinal homeostasis and therefore IBD pathogenesis. Their normal physiological functions provide a framework for understanding how dysregulation of exosome secretory and regulatory pathways may contribute to disease progression. Advances in exosome bioengineering are also expanding what exosomes can do, allowing for improved targeting, enhanced gastrointestinal stability, and more efficient loading of therapeutic cargo. These developments suggest that exosome-based therapies could become valuable additions to existing IBD treatments, especially for patients who do not respond well to conventional drugs. At the same time, questions remain. Their heterogeneous nature and source-dependent differences in cargo profiles provide unique challenges. Further, their complex interactions with and within the gastrointestinal tract are only beginning to be understood. As such, future progress will depend on bridging mechanistic insights with translational work. In conclusion, this field is clearly important and better understanding of the contribution of exosomes in driving IBD pathogenesis could reshape how IBD is managed.
